# Variations in right colic vascular anatomy observed during laparoscopic right colectomy

**DOI:** 10.1186/s12957-019-1561-4

**Published:** 2019-01-12

**Authors:** Chuying Wu, Kai Ye, Yiyang Wu, Qiwei Chen, Jianhua Xu, Jianan Lin, Wengui Kang

**Affiliations:** Department of Tumor Surgery, Second Affiliated Hospital of Fujian Medical University, Quanzhou, People’s Republic of China

**Keywords:** Laparoscope, Right colon radical resection, Vascular anatomic variation, Operative management of vessels

## Abstract

**Background:**

This study aimed to analyze right colonic vascular variability.

**Methods:**

The study included 60 consecutive patients who underwent laparoscopic radical right colectomy and D3 lymph node dissection for malignant colonic cancer on the ileocecal valve, ascending colon or hepatic flexure (March 2013 to October 2016). The videos of the 60 surgical procedures were collected. Variations of right colonic vascular anatomy were retrospectively analyzed based on 60 high-resolution surgical videos of laparoscopic surgery.

**Results:**

The superior mesenteric artery and vein were present in all cases; 95.0% (57/60) had the superior mesenteric artery on the left side of the superior mesenteric vein. The ileocolic artery and vein occurred in 96.7% (58/60) and 100% (60/60) of cases, respectively; 50.0% (29/58) had the ileocolic artery passing the superior mesenteric vein anteriorly. Thirty-three (55.0%) cases had a right colic artery, and 2 (3.33%) had a double right colic artery; 90.9% (30/36) had the right colic vein passing anterior to the superior mesenteric artery. Fifty-six (93.3%) cases had a right colic vein; 7 (12.5%) had a right colic vein accompanied by a right colic artery, 66.1% (37/56) had the right colic vein draining into the gastrocolic trunk of Henle, 23.2% (13/56) had the right colic vein directly draining into superior mesenteric vein, and 10.7% (6/56) had one right colic vein draining into the superior mesenteric vein and the other into the gastrocolic trunk of Henle. Fifty-three (88.3%) cases had a gastrocolic trunk of Henle: a gastrocolic trunk in 35.8% (19/53), a gastropancreatic trunk in 9.4% (5/53), and a gastropancreaticocolic trunk in 54.7% (29/53). The frequencies of middle colic artery and vein were respectively 100% (60/60) and 93.3% (56/60).

**Conclusions:**

Right colonic vascular variations were classified in Chinese patients. Notable findings included a superior mesenteric artery positioned to the right of the superior mesenteric vein and variation in middle colic artery length. This knowledge may be helpful to colorectal surgeons and could potentially help to improve safety by reducing vascular complications during minimally invasive procedures.

## Background

Laparoscopic surgery for colon cancer [1] has been extensively validated [2], and long-term survival outcomes are comparable between laparoscopic and open surgery [3]. Laparoscopic right colectomy based on complete mesocolic excision [4, 5] increases the number of dissected lymph nodes, improves prognosis, and lowers local relapse [6, 7]. Nevertheless, anatomic variations of the right colon vasculature are complex [8], and improper management of vessels during laparoscopic surgery can cause vascular complications [9]. Studies of right colon vascular variations could potentially minimize the risk of complications. Most previous autopsy and imaging studies of the right colon have focused on arteries [10–13] rather than veins, although venous tributaries of the right colon were recently described in a Japanese cohort using three-dimensional (3D) computed tomography (CT) [14]. Nonetheless, variations of right colon vascularity in Chinese people remain uncharacterized. Preoperative 3D-CT vascular reconstruction is not performed routinely due to radiation risks and other considerations [15, 16].

We consider that a detailed knowledge of right colon vascular variations is important to improve safety and reduce the risks of vascular complications during minimally invasive surgery. This study aimed to retrospectively review high-resolution videos of laparoscopic radical right colectomy (LRRC) and explore right colon vascular variations in order to summarize the patterns of the variations and identify methods of coping with these variations during surgery. It was anticipated that our findings would provide a useful reference for surgeons.

## Methods

### Study design

This retrospective study enrolled patients who underwent LRRC at the Department of Tumor Surgery, Second Affiliated Hospital of Fujian Medical University (March 2013 to October 2016). The local ethics committee approved the study. All procedures were in accordance with ethical standards of the relevant committee on human experimentation and Helsinki Declaration. Informed consent was waived because the study was retrospective.

Consecutive patients were initially enrolled based on the following inclusion criteria: (1) malignant colonic neoplasm clinically or pathologically diagnosed before surgery; (2) tumor on the ileocecal valve, ascending colon or hepatic flexure (distal end < 10 cm from the transverse colon); (3) LRRC performed between March 2013 and October 2016; (4) standard D3 lymph node dissection undertaken according to the complete mesocolic excision principle. The following exclusion criteria were then applied: (1) artery-vein classifications or compositions unrecognized due to ineligible standards for D3 lymph node dissection (one case); (2) videos unclear, incomplete, or damaged (five cases); (3) impossible to distinguish artery-vein classifications/compositions due to excessive hemorrhage/blurred operative fields (three cases); (4) palliative rather than radical resection performed (five cases). Based on the inclusion and exclusion criteria, the final analysis included 60 patients.

### Surgical approach

The videos of all 60 surgical procedures featured LRRC (or extended LRRC) using a standard five-port medial approach [17, 18]. The central-to-medial approach was used with the patient in a dorsal elevated, left-tilted position. The small intestine and mesentery were positioned in the left lower abdomen to expose the visual field. The superior mesenteric vein was used as a landmark for the medial border of the LRRC. The mesocolon-mesoileum junction was cut at the inferior ileocolic margin. Via this window, Toldt’s gap was extended to expose the horizontal/descending part of the duodenum. The superior mesenteric vein was divided up to the inferior pancreatic margin. The ileocolic artery and right colic artery anterior to the superior mesenteric vein were identified and clamped/transected. The right posterior colon space was extended superiorly, and the superior pancreaticoduodenal vein, right colic vein, and gastrocolic trunk of Henle were identified. The superior mesenteric vein was clamped/transected. The middle colic artery was identified at the pancreatic neck. For extended LRRC, the middle colic artery was clamped/transected at the root, and complete lymph node dissection was performed. For standard LRRC, the left branch of the middle colic artery was preserved. The middle colic vein, identified after opening the superior mesenteric vein sheath, was clamped/transected. The gastrocolic trunk of Henle and tributaries were identified, and every branch was divided. For standard LRRC, the anterior superior pancreaticoduodenal vein was preserved while the right gastro-omental vein, right colic (and accessory right colic) vein, and middle colic vein were transected. For extended LRRC, the gastrocolic trunk of Henle was clamped/transected at its root.

The transverse colon was repositioned after division of the lower colon. For standard LRRC, the gastrocolic ligament was divided outside the gastro-omental arch, Toldt’s gap was opened between the mesogastrium and transverse mesocolon, and the transverse mesocolon anterior lobe was opened from the inferior pancreatic margin to the inferior region of the colon. For extended LRRC, group IV lymph nodes and lymphoid adipose tissue within 10 cm of the pyloric arch were removed. The right gastro-omental artery was identified and transected.

The patient was adjusted to the Trendelenburg position and left-tilted 15 °. The ileocecus, ascending colon, and hepatic flexure were divided, starting from the “yellow-white boundary” of the ileocecus, mesostenium root, and paracolic sulci up to the interior right posterior colic space. The right colon and accessory fixation tissues were then completely divided.

The pneumoperitoneum was closed, and the right colon (including tumor, mesocolon, and sufficient intestinal segment) was removed via a 5-cm median incision in the upper abdomen. End-to-side (or side-to-side) anastomosis of the ileum and transverse colon was performed. The free margins of the mesoileum and transverse mesocolon were sutured in a closed or open state.

### Study methods

All operations were completed by one group of surgeons (a director, associate director, attending surgeon, and resident surgeon). The surgeons understood the vascular anatomic variations of the right colon and could perform all the procedures proficiently. The videos were thoroughly reviewed after surgery. The anatomic characteristics and spatial relationships of the vessels were analyzed, with annotation of screenshots from the videos and collection of results in tables. Based on our experience, we discussed measures to avoid operative vascular complications.

### Statistical analysis

SPSS 19.0 (SPSS Inc., Chicago, IL, USA) was used for statistical analysis. Enumeration data are expressed as mean ± standard deviation.

## Results

### Patient characteristics

Sixty patients (aged 23–83 years; 33 males) were included (Table 1). The tumor locations and perioperative data are summarized in Table 1.

**Table 1:** Baseline and perioperative data

Variable	Data
Age, mean ± SD (range), years	58.5 ± 13.4 (23–83)
Sex, *n*(%)	
Men	33(55)
Women	27(45)
BMI, mean ± SD, kg/m^2^	21.8 ± 2.6
ASA score, *n*(%)	
0	47(78.3)
0.01	13(21.7)
Previous abdominal surgery history, *n*(%)	10(16.7)
Tumor location, *n*(%)	
Cecum	10(16.7)
Ascending colon	43(71.6)
Hepatic flexure	6(10)
Appendix	1(1.7)
Operation time, mean ± SD, min	154.1 ± 17.6
Estimated blood loss, mean ± SD, mL	91.2 ± 103.9
Length of hospital stay, mean ± SD (range), days	4.7 ± 7.2 (14–56)
Complications, *n*(%)	24
Ileus	13(54.2)
Urinary retention	0(0)
Wound problem	2(8.3)
Postoperative bleeding	4(16.7)
Others (cholangitis or pneumonia)	6(25)
Operative mortality, *n*(%)	0(0)
Tumor size, mean ± SD, cm	5.6 ± 2.1
Tumor type, *n*(%)	
Adenocarcinoma	41(68.3)
Mucinous adenocarcinoma	9(15)
Other	10(16.7)
Histologic grade, *n*(%)	
W/d	3(5)
M/d	41(68.3)
P/d	9(15)
Others	7(11.7)
Stage, *n*(%)	
0	10(16.7)
1	3(5)
2	25(41.7)
3	18(30)
4	4(6.7)
No. of metastatic LNs, mean ± SD (range)	1.7 ± 3.6 (0–20)
No. of harvested LNs, mean ± SD (range)	34.6 ± 13.8 (4–67)
Resection margin, mean ± SD (range), cm	
Proximal	11.7 ± 3.8 (5.5–23.5)
Distal	11.6 ± 4.9 (2.2–26)

### Variations in right colic vascular anatomy

**Fig. 1:**
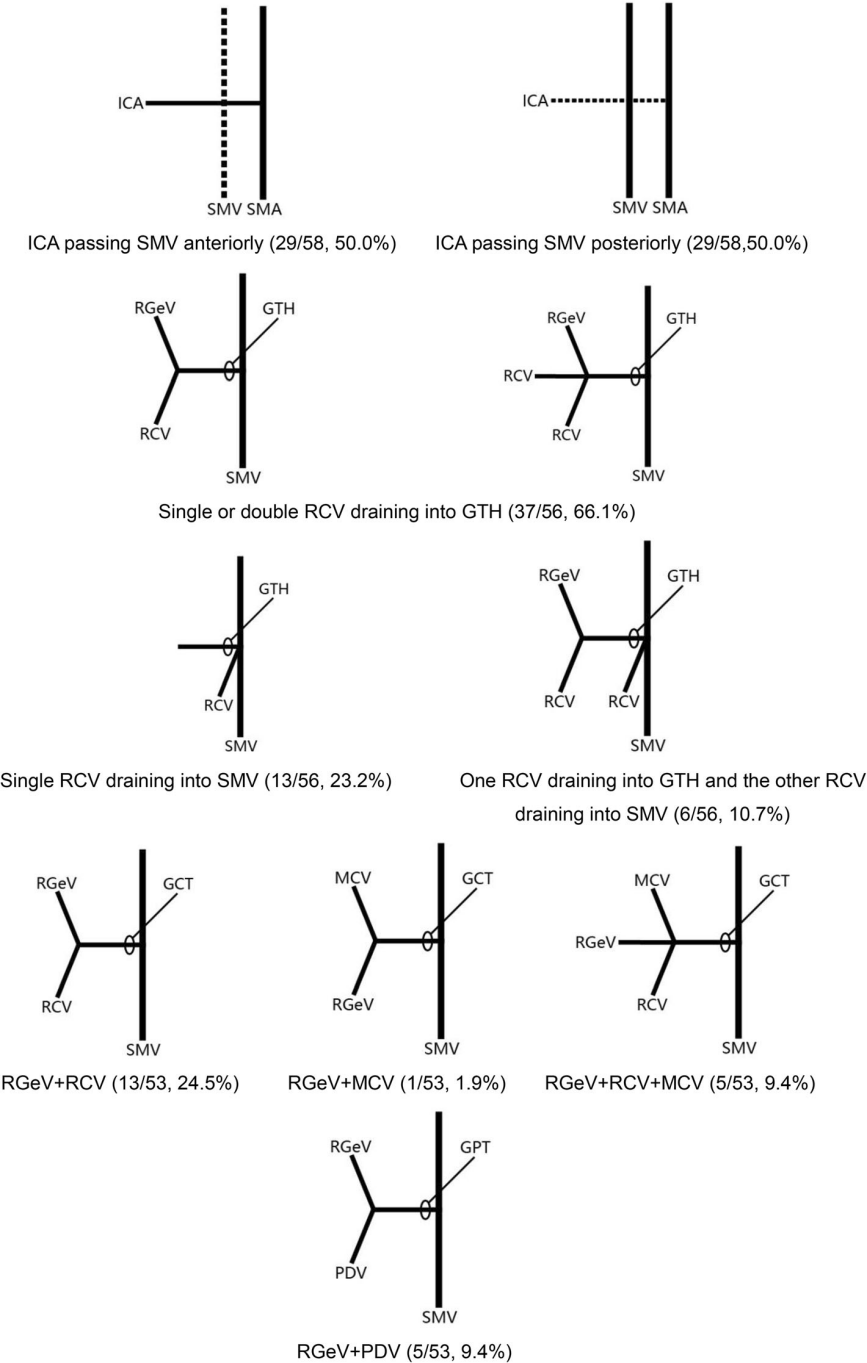
Variations of right colic vascular vessels. The number of cases and percentages for each anatomic variation are shown. GCT, gastrocolic trunk; GPT, gastropancreatic trunk; GTH, gastrocolic trunk of Henle; ICA, ileocolic artery; MCV, middle colic vein; PDV, pancreaticoduodenal vein; RCV, right colic vein; RGeV, right gastro-omental vein; SMA, superior mesenteric artery; SMV, superior mesenteric vein

The variations in right colic vascular anatomy observed in the 60 patients are summarized in Table 2 and Fig. 1. Specific aspects of the anatomic variations are described below.

**Table 2:** Variations of right colic vascular vessels

Anatomic feature	*n*(%)
Relative spatial relationship of ICA with SMV	
ICA passing SMV anteriorly	29(50.0%)
ICA passing SMV posteriorly	29(50.0%)
RCV drainage into superior veins	
Single or double RCV draining into GTH (one patient had double RCV draining into GTH)	37(66.1%)
Single RCV draining into SMV	13(23.2%)
One RCV draining into GTH and the other RCV draining into SMV	6(10.7%)
Constituent tributaries of GTH	
GCT	
RGeV, RCV	13(24.5%)
RGeV, MCV	1(1.9%)
RGeV, RCV, MCV	5(9.4%)
GPT	
RGeV, PDV	5(9.4%)
GPCT	
RGeV, PDV, RCV	14(26.4%)
RGeV, PDV, MCV	4(7.5%)
RGeV, PDV, double RCV	1(1.9%)
RGeV, PDV, RCV, MCV	10(18.9%)
The distance between MCA bifurcation and the starting point	
≤ 1 cm	8(15.7%)
1–2 cm	34(66.7%)
> 2 cm	9(17.6%)
MCV drainage into superior vein	
MCV draining into GTH	20(35.7%)
MCV draining into SMV	
Double RCV draining into SMV in one patient	27(48.2%)
One MCV draining into GTH and the other into SMV	7(12.5%)
One MCV draining into GTH and the other two into SMV	2(3.6%)

*GCT* gastrocolic trunk; *GPCT* gastropancreaticocolic trunk; *GPT* gastropancreatic trunk; *GTH* gastrocolic trunk of Henle; *ICA* ileocolic artery; *MCA* middle colic artery; *MCV* middle colic vein; *PDV* pancreaticoduodenal vein; *RCV* right colic vein; *RGeV* right gastro-omental vein; *SMV* superior mesenteric vein

### Superior mesenteric artery and superior mesenteric vein

**Fig. 2:**
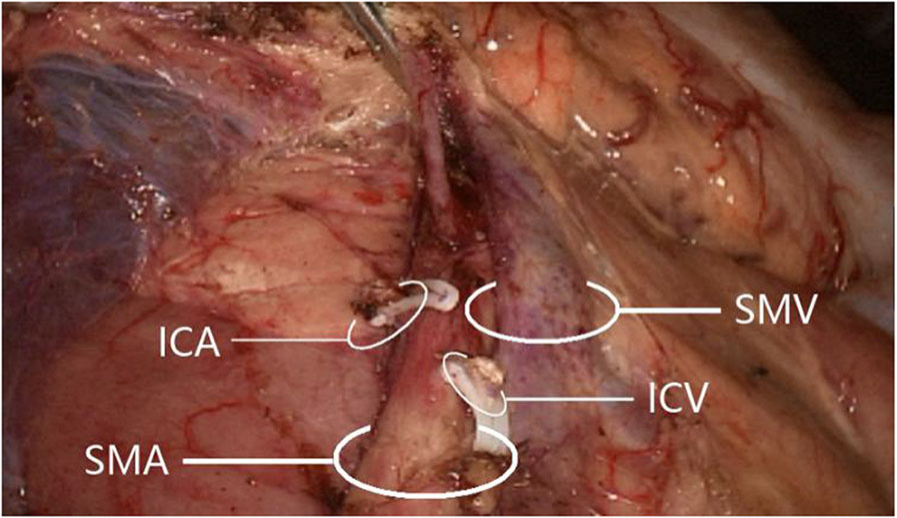
Superior mesenteric artery on the right side of superior mesenteric vein. ICA, ileocolic artery; ICV, ileocolic vein; SMA, superior mesenteric artery; SMV, superior mesenteric vein

The superior mesenteric artery and superior mesenteric vein were present in all 60 cases. The superior mesenteric artery was left of the superior mesenteric vein in 57 cases (95.0%) and right of the superior mesenteric vein in 3 cases (5.0%) (Fig. 2).

### Ileocolic artery and ileocolic vein

**Fig. 3:**
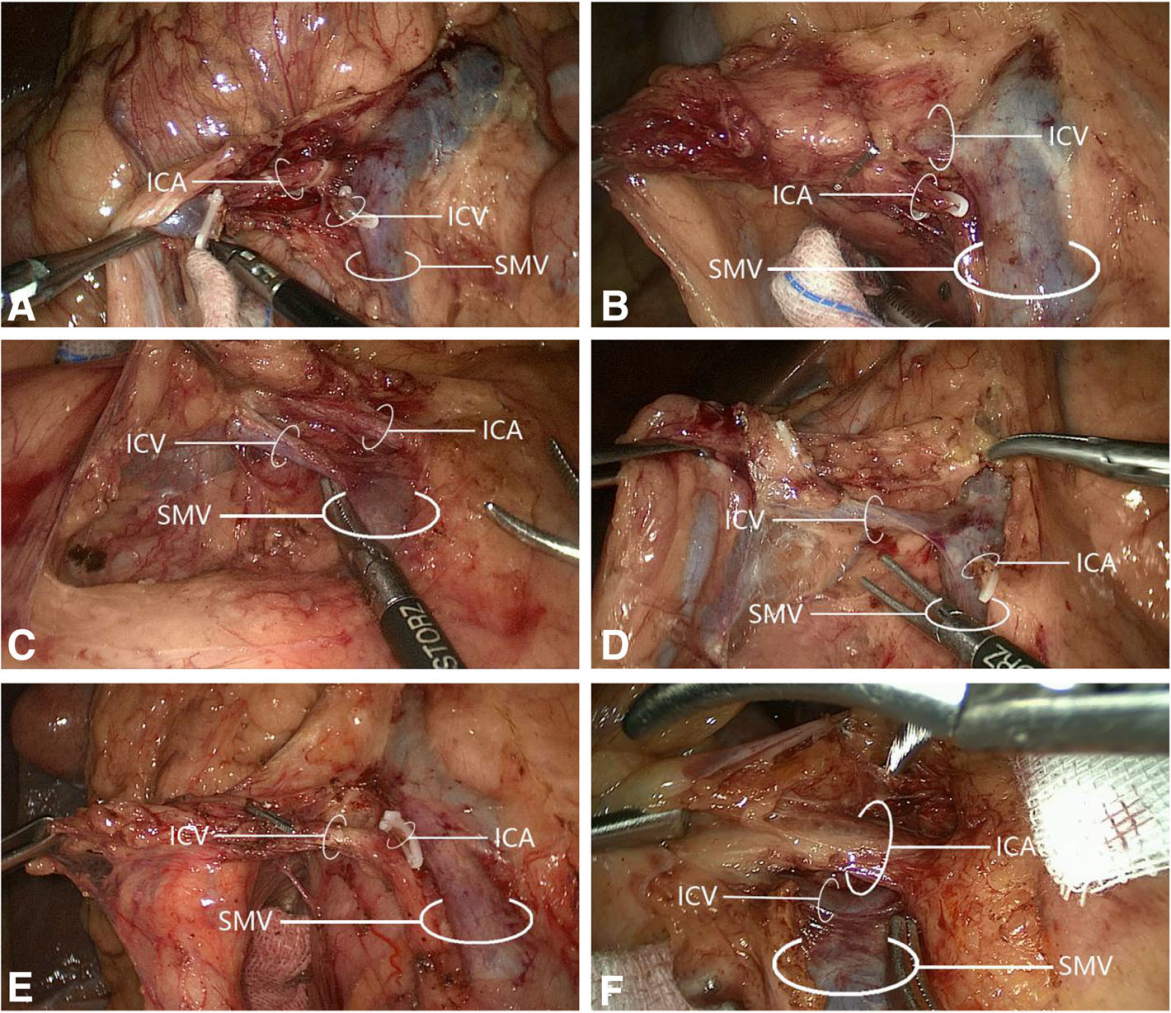
The relative spatial relationships of the ileocolic artery with the ileocolic vein. The ileocolic artery was **a** posterior superior to the ileocolic vein; **b** posterior inferior to the ileocolic vein; **c** anterior superior to the ileocolic vein; **d** anterior inferior to the ileocolic vein; **e** posterior medial to the ileocolic vein; and **f** anterior medial to the ileocolic vein. ICA, ileocolic artery; ICV, ileocolic vein; SMV, superior mesenteric vein

The ileocolic artery was present in 58/60 cases (96.7%), and the ileocolic vein was present in 60 cases (100%); their spatial relationships are displayed in Fig. 3 and Table 2. Among 58 cases with an ileocolic artery, its positioning relative to the ileocolic vein was anteromedial in 5 (8.6%), anterosuperior in 18 (31.0%), anteroinferior in 6 (10.3%), posteromedial in 4 (6.9%), posterosuperior in 19 (32.8%), and posteroinferior in 6 (10.3%).

### Right colic artery and right colic vein

**Fig. 4:**
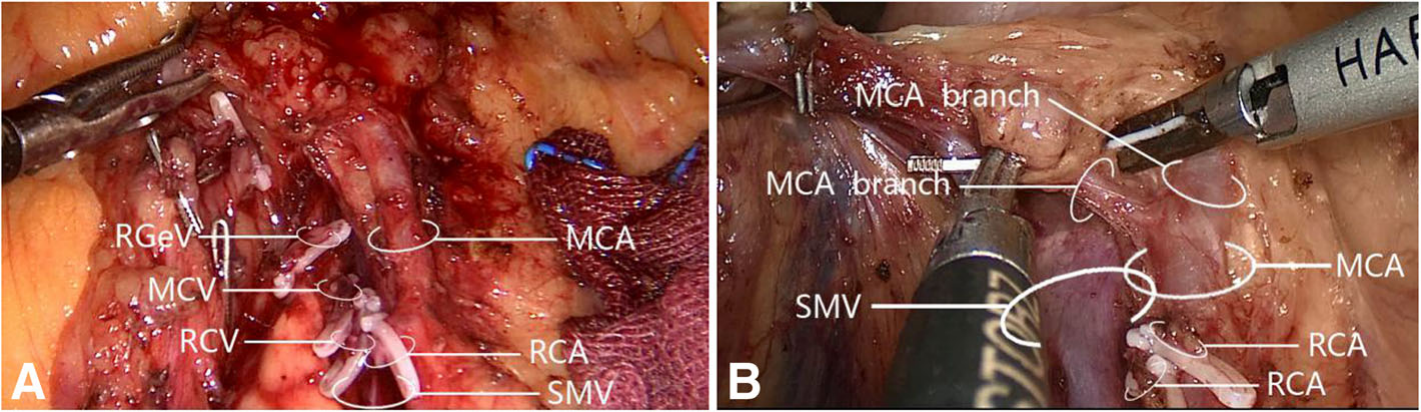
The common trunk of right colic artery and middle colic artery draining into superior mesenteric artery. **a** The common trunk of one right colic artery and middle colic artery draining into a superior mesenteric artery. **b** The common trunk of a double right colic artery and one middle colic artery draining into a superior mesenteric artery. MCA, middle colic artery; MCV, middle colic vein; RCA, right colic artery; RCV, right colic vein; RGeV, right gastro-omental vein; SMV, superior mesenteric vein

**Fig. 5:**
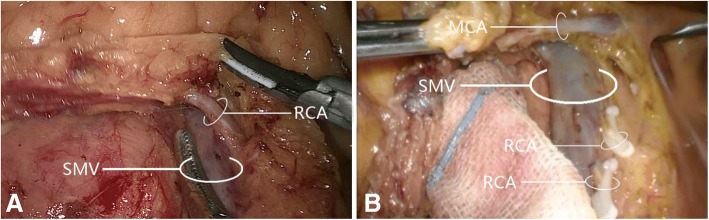
The relative spatial relationships of the right colic artery with superior mesenteric vein. **a** One right colic artery passing superior mesenteric vein anteriorly. **b** Double right colic artery passing superior mesenteric vein anteriorly. MCA, middle colic artery; RCA, right colic artery; SMV, superior mesenteric vein

The right colic artery was present in 33/60 cases (55.0%). Among these 33 patients, 31 (93.9%) had one right colic artery, with 8 (24.2%) having a common trunk for the right colic artery and middle colic artery that drained into the superior mesenteric artery (Fig. 4); the remaining 2 patients (6.1%) had a double right colic artery. Analysis of the relationship between the right colic artery and superior mesenteric vein (Fig. 5) revealed that 30/33 cases (90.9%) had a single/double right colic artery anterior to the superior mesenteric vein, while 3 (9.1%) had the superior mesenteric artery to the right of the superior mesenteric vein (i.e., no spatial relationship between the right colic artery and superior mesenteric vein); no cases had the right colic artery posterior to the superior mesenteric vein.

**Fig. 6:**
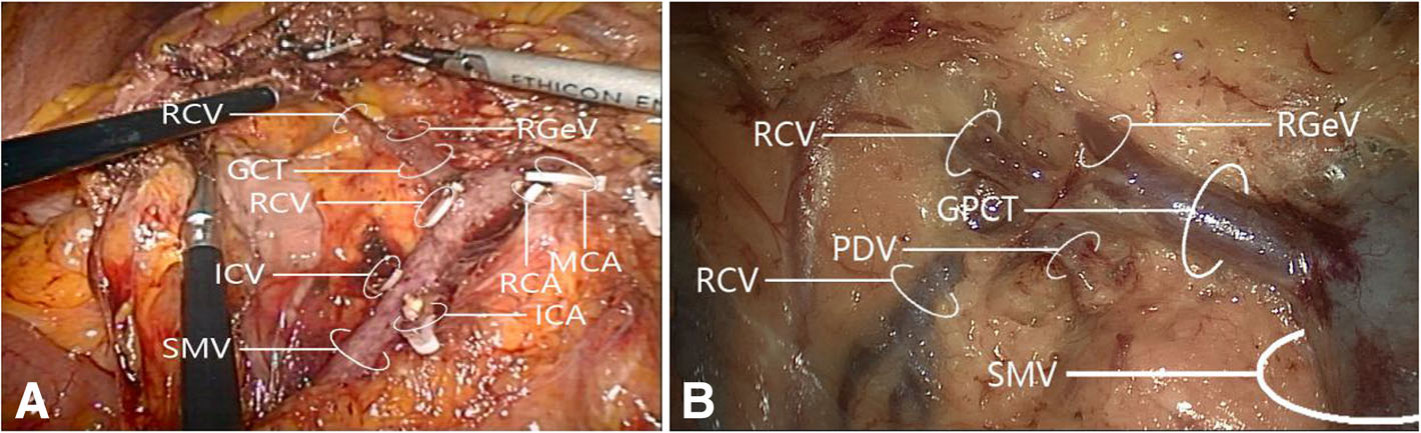
Drainage of double right colic vein into superior veins. **a** One right colic vein draining into superior mesenteric vein and the other draining into the gastrocolic trunk of Henle. **b** Both right colic veins draining into the gastrocolic trunk of Henle. GCT, gastrocolic trunk; GPCT, gastropancreaticocolic trunk; ICA, ileocolic artery; MCA, middle colic artery; PDV, pancreaticoduodenal vein; RCA, right colic artery; RCV, right colic vein; RGeV, right gastro-omental vein; SMV, superior mesenteric vein

The right colic vein was present in 56/60 cases (93.3%): 49 (87.5%) had one right colic vein and 7 (12.5%) had a double right colic vein. Among 49 cases with one right colic vein, 7 (14.3%) had a right colic vein accompanied by a right colic artery. Right colic vein drainage was further examined (Table 2). Among 49 cases with one right colic vein, the right colic vein drained into the gastrocolic trunk of Henle in 36 (73.5%) and superior mesenteric vein in 13 (26.5%). Among 7 cases with a double right colic vein, 6 (85.7%) had one right colic vein draining into the superior mesenteric vein and the other into the gastrocolic trunk of Henle, and 1 (14.3%) had a double right colic vein draining into the gastrocolic trunk of Henle; there were no instances of a double right colic vein draining into the superior mesenteric vein (Fig. 6).

### Gastrocolic trunk of Henle

**Fig. 7:**
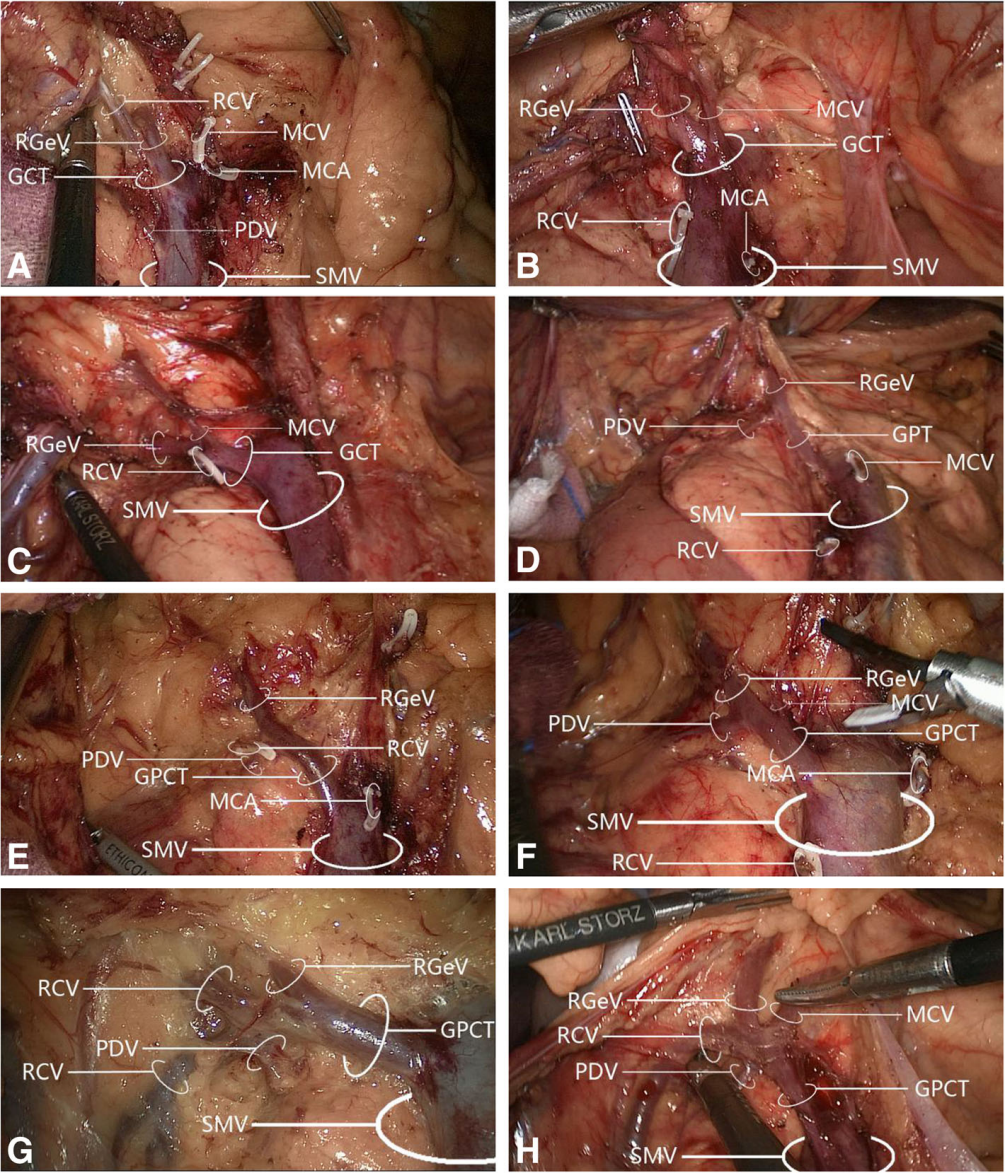
Variations in gastrocolic trunk of Henle tributary constituents. **a** Gastrocolic trunk-type composed of two tributaries: right gastro-omental vein and right colic vein. **b** Gastrocolic trunk-type composed of two tributaries: right gastro-omental vein and middle colic vein. **c** Gastrocolic trunk-type composed of three tributaries: right gastro-omental vein, right colic vein, and middle colic vein. **d** Gastropancreatic trunk-type composed of two tributaries: right gastro-omental vein and pancreaticoduodenal vein. **e** Gastropancreaticocolic trunk-type composed of three tributaries: right gastro-omental vein, right colic vein, and pancreaticoduodenal vein. **f** Gastropancreaticocolic trunk-type composed of three tributaries: right gastro-omental vein, middle colic vein, and pancreaticoduodenal vein. **g** Gastropancreaticocolic trunk-type composed of four tributaries: right gastro-omental vein, two right colic veins, and pancreaticoduodenal vein. **h** Gastropancreaticocolic trunk-type composed of four tributaries: right gastro-omental vein, right colic vein, middle colic vein, and pancreaticoduodenal vein. GCT, gastrocolic trunk; GPT, gastropancreatic trunk; GPCT, gastropancreaticocolic trunk; MCA, middle colic artery; MCV, middle colic vein; PDV, pancreaticoduodenal vein; RCV, right colic vein; RGeV, right gastro-omental vein; SMV, superior mesenteric vein

**Fig. 8:**
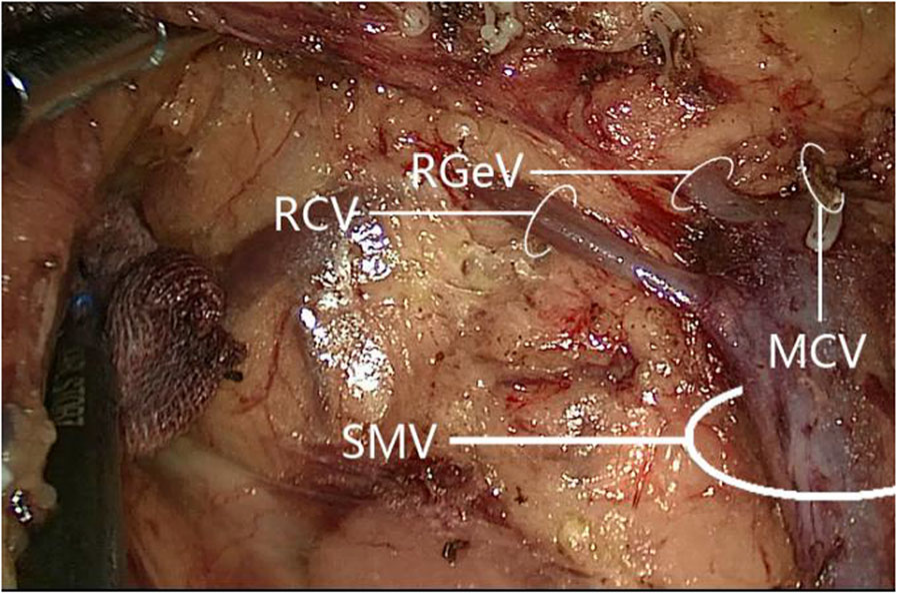
In the absence of a gastrocolic trunk of Henle, the right gastro-omental vein drained into the superior mesenteric vein. MCV, middle colic vein; RCV, right colic vein; RGeV, right gastro-omental vein; SMV, superior mesenteric vein

A gastrocolic trunk of Henle with four branches (right gastro-omental vein, right colic vein, middle colic vein, and pancreaticoduodenal vein) was present in 53/60 cases (88.3%). Variations in the constituent tributaries of the gastrocolic trunk of Henle included gastrocolic trunk-type in 19 cases (35.8%), composed of 2 or 3 tributaries of the right gastro-omental vein and right colic vein without the middle colic vein; gastropancreatic trunk-type in 5 cases (9.4%), composed of 2 tributaries of the right gastro-omental vein and anterior superior pancreaticoduodenal vein; gastropancreaticocolic trunk-type in 29 cases (54.7%), composed of 3 or 4 tributaries of the right gastro-omental vein, anterior superior pancreaticoduodenal vein, right colic vein and middle colic vein; and gastrocolic trunk of Henle absence (i.e., right gastro-omental vein draining directly into the superior mesenteric vein) in 7 cases (13.2%). Detailed information is provided in Figs. 7 and 8 and Table 2.

### Middle colic artery and middle colic vein

**Fig. 9:**
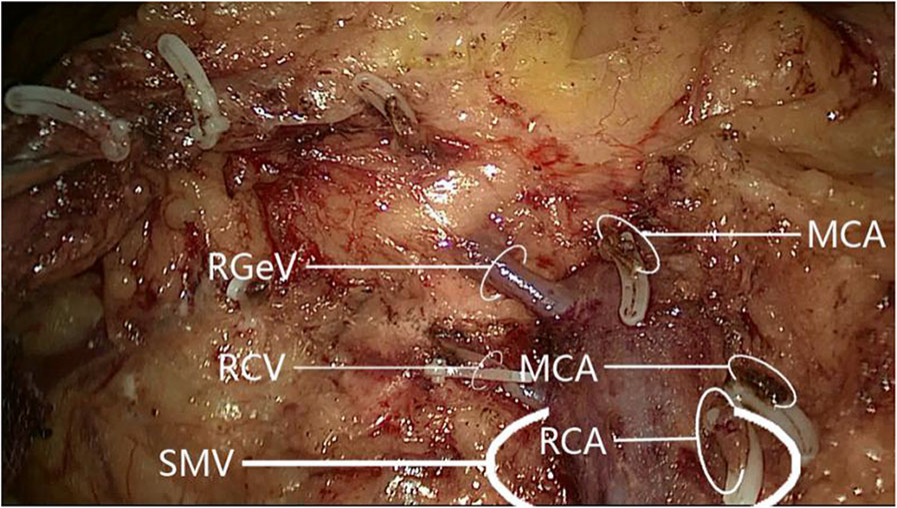
The presence of double middle colic artery. MCA, middle colic artery; RCA, right colic artery; RCV, right colic vein; RGeV, right gastro-omental vein; SMV, superior mesenteric vein

The middle colic artery was present in all 60 cases (100%); 8 (13.3%) had a common trunk for the middle colic artery and right colic artery (Fig. 4), while 1 (1.7%) had a double middle colic artery (Fig. 9). Among 51 cases with one middle colic artery (excluding those with common middle colic artery/right colic artery trunk or double middle colic artery), the distance of the middle colic artery bifurcation from the pancreatic neck was ≤ 1 cm in 8 (15.7%), 1–2 cm in 34 (66.7%), and > 2 cm in 9 (17.6%). Morphologic features of the middle colic artery are shown in Table 2.

**Fig. 10:**
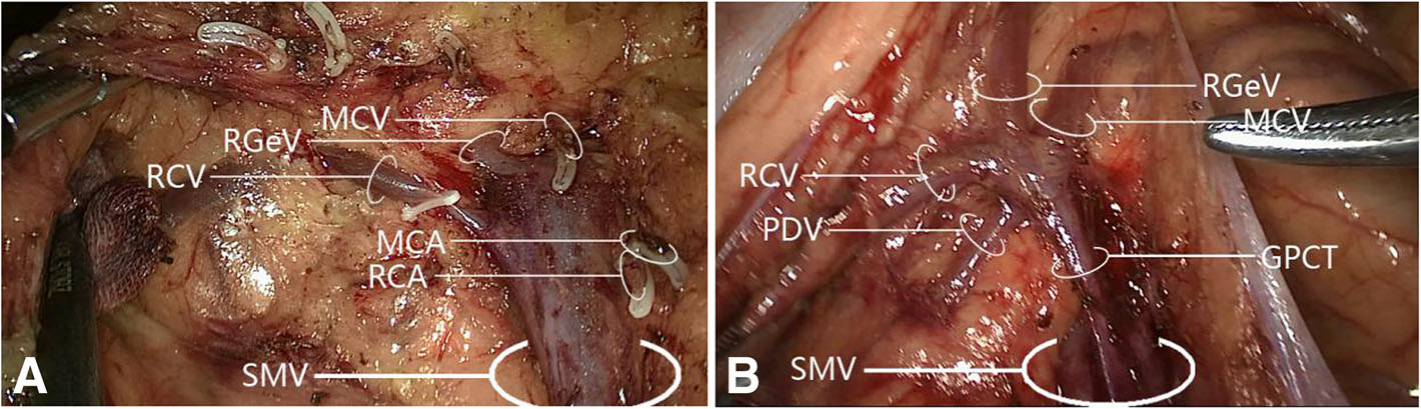
Drainage of sole middle colic vein into superior veins. **a** Middle colic vein draining into the superior mesenteric vein. **b** Middle colic vein draining into the gastrocolic trunk of Henle. GPCT, gastropancreaticocolic trunk; MCA, middle colic artery; MCV, middle colic vein; PDV, pancreaticoduodenal vein; RCA, right colic artery; RCV, right colic vein; RGeV, right gastro-omental vein; SMV, superior mesenteric vein

**Fig. 11:**
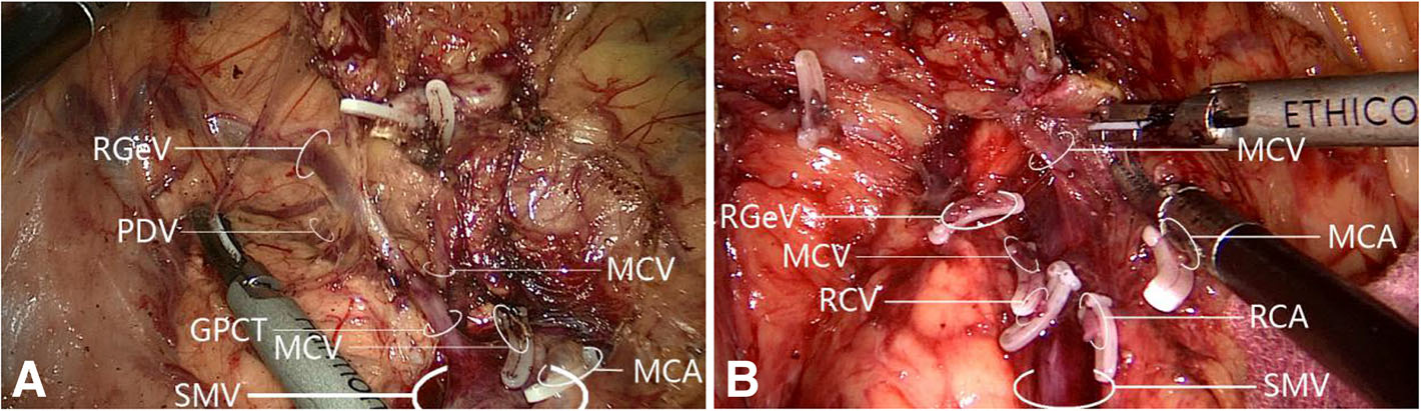
Drainage of double middle colic vein into superior veins. **a** One middle colic vein draining into the superior mesenteric vein, and the other draining into the gastrocolic trunk of Henle. **b** Both middle colic veins draining into the superior mesenteric vein. No cases had both middle colic veins draining into the gastrocolic trunk of Henle. GPCT, gastropancreaticocolic trunk; MCA, middle colic artery; MCV, middle colic vein; PDV, pancreaticoduodenal vein; RCA, right colic artery; RCV, right colic vein; RGeV, right gastro-omental vein; SMV, superior mesenteric vein

**Fig. 12:**
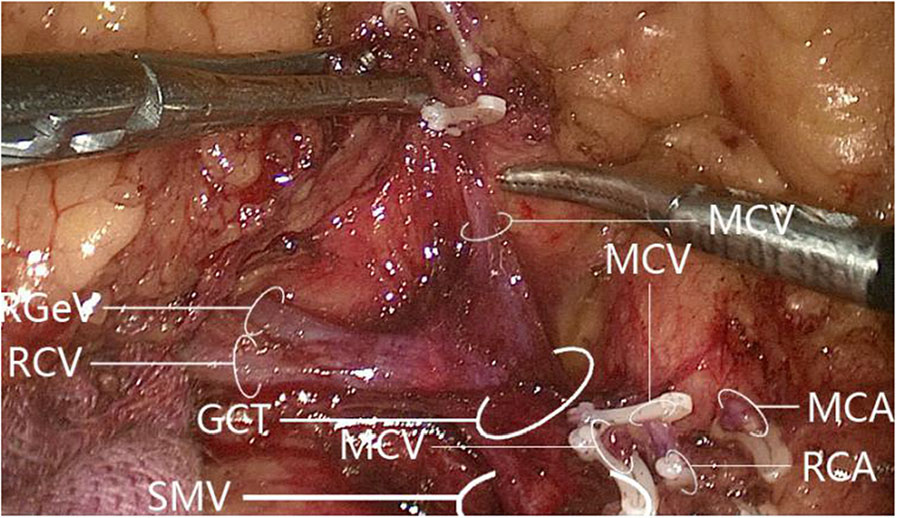
Drainage of triploid middle colic vein into superior veins. One middle colic vein drained into the gastrocolic trunk of Henle while the other two middle colic veins drained into the superior mesenteric vein. GCT, gastrocolic trunk; MCA, middle colic artery; MCV, middle colic vein; RCA, right colic artery; RCV, right colic vein; RGeV, right gastro-omental vein; SMV, superior mesenteric vein

The middle colic vein was present in 56/60 cases (93.3%): 45 (80.4%) had one middle colic vein, 9 (16.1%) had a double middle colic vein, and 2 (3.6%) had a triploid middle colic vein. Among 45 cases with one middle colic vein, it drained into the superior mesenteric vein in 25 (55.6%) and gastrocolic trunk of Henle in 20 (44.4%) (Fig. 10). Among 9 cases with a double middle colic vein, 7 (77.8%) had one middle colic vein draining into the superior mesenteric vein and the other into the gastrocolic trunk of Henle, while 2 (22.2%) had both middle colic veins draining into the superior mesenteric vein; no cases had both middle colic veins draining into the gastrocolic trunk of Henle (Fig. 11). Both cases with triploid middle colic vein had one middle colic vein draining into the gastrocolic trunk of Henle and two draining into the superior mesenteric vein (Fig. 12).

## Discussion

The anatomy of the right colonic vessels varies greatly. Handling the relevant blood vessels, especially Henle’s branch, during LRRC for right colon cancer is highly challenging, and damage to the vessels is a major cause of intraoperative bleeding and conversion to other surgical treatments. Previous research has reported vascular variations of the right colon and highlighted their importance, and it is recognized that failure to identify vascular variations during minimally invasive surgery can lead to troublesome bleeding [19]. However, consensus is lacking regarding the assessment and management of these variations. The objectives of this retrospective analysis were to characterize right colon vascular variations from video recordings obtained during LRRC and summarize the patterns in these variations. Our results highlight variations in the branches of the right colonic blood vessels and the relationships between these branches. Our study describes two rarely reported variations in right colon vasculature, namely, a superior mesenteric artery positioned to the right of the superior mesenteric vein and variation in middle colic artery length. Although several published studies have described variations of the right colonic vascular anatomy, our new findings extend our knowledge in this area and highlight uncommon features that might be encountered by surgeons performing laparoscopic right colectomy. We anticipate that knowledge of the right colon vascular variations detailed in this study will help surgeons to reduce the risk of vascular complications during minimally invasive surgery, thereby improving safety and potentially reducing hospitalization time and costs of treatment.

Preoperative 3D-CT has been reported to have high sensitivity, specificity, accuracy, and reliability in establishing mesenteric vessel anatomy [20]. Nonetheless, false-negative and false-positive CT findings do occur [20]. Furthermore, preoperative 3D-CT is not used routinely to define right colonic vascular anatomy due to radiation risks and other factors [15, 16]. The availability of video recordings obtained during LRRC at our institution enabled us to carry out a retrospective analysis of the anatomic variations of the right colonic blood vessels with greater accuracy that 3D-CT and without exposing the patients to radiation. An additional advantage of our approach is that it helped to identify methods that could be used to cope with these variations during minimally invasive surgery. In particular, our research provides insights regarding how to identify, expose, and dissect right colonic blood vessels during surgery in order to minimize the risk of damage to these blood vessels and thus avoid troublesome bleeding. The findings of this study could help surgeons to better understand the different surgical planes, anatomic spaces, and right colonic vascular variations that might be encountered during surgery. In turn, this could facilitate more skillful management of vascular variations during the operation. We speculate that the key approach to dealing with right colonic vascular variations is to track the ileocolonic vessel and Henle’s branch along the superior mesenteric vein and prioritize dissection of Henle’s branch and other branches. Insights into vascular anatomy provided by our study and others like it could improve the quality of the LRRC procedure, enhance the safety of the operation, and accelerate the postoperative recovery of the patient.

The superior mesenteric artery is usually located left of the superior mesenteric vein [21], but we observed it right of the superior mesenteric vein in 3/60 patients. This uncommon finding has only been reported in a small number of earlier studies. Menten et al. used imaging techniques to identify one case of a superior mesenteric artery to the right of the superior mesenteric vein among 80 children with a normal duodenum position, and two additional cases were described among three children with an abnormal duodenum position [22]. A superior mesenteric artery to the right of the superior mesenteric vein was also demonstrated by imaging in an adult patient with malrotation [23]. In addition, Sodhi et al. reported a counterclockwise (rather than clockwise) rotation of the superior mesenteric vein around the superior mesenteric artery in a minority of asymptomatic children [24].

Previous investigations [10, 25, 26] found that the ileocolic artery, right colic artery, and middle colic artery sprouted individually from the superior mesenteric artery in 10.7–45.0% of cases, the ileocolic artery and middle colic artery were both present in 100%, and the right colic artery sprouted independently from the superior mesenteric artery in 19–45%. The superior mesenteric vein is a key landmark for right colic vascular anatomy [27] and in our study was identifiable as a purple-blue bulging vertical cord.

The ileocolic artery was present in 96.7% of our cases, i.e., not invariably present as suggested before [14, 28]. The ileocolic artery was anterior to the superior mesenteric vein in 50.0% of our cases, higher than values (33.0–36.7%) reported previously [11, 21]. Since the ileocolic vascular pedicle was invariably present in our study, it is reasonable to take its intersection with the superior mesenteric vein as the starting point for LRRC. The spatial relationship between the ileocolic artery and ileocolic vein was mostly anterior-posterior, and most ileocolic veins drained directly into the superior mesenteric vein with only a few joining the right colic vein before draining into the gastrocolic trunk of Henle. We suggest the ileocolic vein be managed before the ileocolic artery because it is more prone to tearing. In view of the variable spatial relationships, a reasonable approach is to incise the mesentery surface 1 cm below where the ileocolic vein drains into the superior mesenteric vein, incise the fat and superior mesenteric vein pedicle, and enter the nonvascular interval of the pedicle to manage the ileocolic vein, prior to extending Toldt’s gap to manage the ileocolic artery.

Excessive positional closeness of the ileocolic artery and gastrocolic trunk of Henle often causes right colic artery absence [29]. The right colic artery was present in 55.0% of our cases and was anterior to the superior mesenteric vein in 90.9% of cases, higher than published values of 19–45% [10, 25, 26] and 62.5–84.2% [11, 21], respectively; indeed, a right colic artery posterior to a superior mesenteric vein is a novel observation. The reasons for these differences are unclear. Notably, two of our patients had a double right colic artery, a feature not reported previously.

Autopsy reports indicate that the right colic vein originates independently from the superior mesenteric vein in 25% of cases [28]. We identified a double right colic vein in 7 cases; 6 had one right colic vein draining into the superior mesenteric vein and the other into the gastrocolic trunk of Henle, validating the concept of the superior right colic vein. The right colic vein and superior right colic vein differed morphologically (one thick and short, the other long and thin) in these 6 cases, although which feature belonged to which vein could not be definitively established.

We suggest that sequential preference should be given to the right colic artery and right colic vein during clamping/transection. The right colic artery and right colic vein are slender vessels whose traction raises the risks of tearing. Since the right colic artery and right colic vein drain directly into the superior mesenteric artery and superior mesenteric vein, respectively, tearing could lead to uncontrollable bleeding, necessitating conversion to open surgery.

The presence of the gastrocolic trunk of Henle and its tributaries is highly variable, but dissection of lymph nodes surrounding the gastrocolic trunk of Henle is critical in view of the metastasis route for hepatic flexure/transverse colon cancer [30]. The gastrocolic trunk of Henle was initially described as formed by the confluence of the right gastro-omental vein and right colic vein, but the pancreaticoduodenal vein [31] and ileocolic vein are also constituent tributaries [14]. The right gastro-omental vein is an inherent constituent tributary [32], and the gastrocolic trunk of Henle is currently recognized as including at least two tributaries, i.e., the right gastro-omental vein plus a colic vein (right colic vein or middle colic vein). In more than half of cases, the gastrocolic trunk of Henle comprises the right gastro-omental vein, right colic vein, and pancreaticoduodenal vein [33]. The gastrocolic trunk of Henle has been classified into three types: gastrocolic trunk, gastropancreatic trunk, and gastropancreaticocolic trunk [34]. The gastrocolic trunk of Henle was present in 88.3% of our cohort, consistent with previous values of 80–100% [27, 31]. The gastrocolic trunk of Henle type was gastrocolic trunk in 35.8%, gastropancreatic trunk in 9.4% and gastropancreaticocolic trunk in 54.7%.

Over-traction/incisional error of the gastrocolic trunk of Henle and tributaries can lead to massive hemorrhage. In this study, the gastrocolic trunk of Henle was observed to drain rightwards into the superior mesenteric vein at 2 cm below the inferior pancreatic margin. The outline of the gastrocolic trunk of Henle and its tributaries could often be identified by observing the distal end of the right colic vein in the posterior space of the transverse colon. When the pancreaticoduodenal vein was preserved, the right gastro-omental vein, right colic vein, and middle colic vein were transected; for an extended right colectomy, the gastrocolic trunk of Henle was clamped at its root and transected.

Reports have varied regarding middle colic artery presence and origin. An autopsy study found the middle colic artery was present in all cases [35], whereas a review of operative data revealed middle colic artery absence in 30%, double middle colic arteries in 32.5%, and triploid middle colic arteries in 6% of patients [36]. The middle colic artery usually sprouts from the superior mesenteric artery but can originate from the hepatic or splenic artery [37] or, rarely, the coeliac trunk [38] or dorsal pancreatic artery [39]. In our study, the middle colic artery originated from the superior mesenteric artery in all cases. We consider these results acceptable because the hepatic artery, splenic artery, and celiac trunk were not routinely dissected during laparoscopic surgery. The middle colic artery was present in all cases, in agreement with previous research [35]. We assume that the middle colic artery and right colic artery split from a common trunk originating from the superior mesenteric artery. Previous research [21] has shown that the middle colic artery generally runs on the left anterior side of the superior mesenteric vein, in agreement with our data. Our results also revealed previously unreported variations in the distance between the left-right bifurcation point and sprouting site (1–2 cm in most cases).

Conventional methods to identify the middle colic artery include dissection of the superior mesenteric vein superiorly from the ileocolic vein (which risks middle colic vein injury, particularly in patients with enlarged vascular root lymph nodes) and rotation of the transverse colon and mesentery (which is less successful in patients with more abdominal fat). We suggest that the middle colic artery can be rapidly recognized using the latter method in patients with lower body mass index. For patients with higher body mass index, cautious use of the former approach helps identify the gastrocolic trunk of Henle and its tributaries while avoiding injury to the middle colic artery and middle colic vein. For the minority of patients with the middle colic artery originating from the superior mesenteric artery as a common trunk with the right colic artery, management should be as described above for the right colic artery, and the left tributary of the middle colic artery should be preserved. When clamping/transecting the middle colic artery, it is important to distinguish between confluence of left/right middle colic artery branches and confluence of the right colic artery and middle colic artery.

The middle colic vein can drain into the splenic vein, jejunal vein, or superior mesenteric vein [14, 28], which are not routinely divided in standard laparoscopic surgery. The apparent absence of the middle colic vein in four of our cases may have been erroneous due to an unrecognized middle colic vein draining into the superior mesenteric vein. Additionally, nine cases in our study had a double middle colic vein and two had a triploid middle colic vein, consistent with previous findings [14]. However, another study of 58 cases identified a double middle colic vein in half the cases and a triploid middle colic vein in 10 cases [28]. The lower number of double and triploid middle colic veins in our study may reflect insufficient anatomic characterization of the left side of the transverse colon.

An important observation was that the middle colic vein was often longer and thinner, and hence more easily missed, if it drained into the superior mesenteric vein rather than the gastrocolic trunk of Henle. Therefore, recognition of vascular variations and careful dissection in the pancreatic neck region are critical.

This study has some limitations. This was a retrospective analysis with a small sample size that considered only patients with right colon cancer. Furthermore, the use of specific inclusion and exclusion criteria may have introduced some selection bias. In addition, the blood vessels of the left colon were not analyzed. Further studies are needed to address these limitations.

## Conclusions

This study provides new insights into the anatomic variations of the right colon vasculature in Chinese patients with colon cancer. In particular, we have identified two rarely reported variations in right colon vasculature: positioning of the superior mesenteric artery to the right of the superior mesenteric vein and variation in middle colic artery length. Understanding these vascular variations will help surgeons performing LRRC to minimize the risks of vascular complications, thereby improving safety and potentially reducing hospitalization time and treatment costs.

## Abbreviations

3D: Three-dimensional
CT: Computed tomography
LRRC: Laparoscopic radical right colectomy

## References

[1] Jemal A, Siegel R, Ward E, Murray T, Xu J, Thun MJ (2007). Cancer statistics, 2007. CA Cancer J Clin.

[2] Juo YY, Hyder O, Haider AH, Camp M, Lidor A, Ahuja N (2014). Is minimally invasive colon resection better than traditional approaches?: first comprehensive national examination with propensity score matching. JAMA Surg.

[3] Lee CZ, Kao LT, Lin HC, Wei PL (2015). Comparison of clinical outcome between laparoscopic and open right hemicolectomy: a nationwide study. World J Surg Oncol.

[4] Hohenberger W, Weber K, Matzel K, Papadopoulos T, Merkel S (2009). Standardized surgery for colonic cancer: complete mesocolic excision and central ligation--technical notes and outcome. Color Dis.

[5] Sondenaa K, Quirke P, Hohenberger W, Sugihara K, Kobayashi H, Kessler H (2014). The rationale behind complete mesocolic excision (CME) and a central vascular ligation for colon cancer in open and laparoscopic surgery : proceedings of a consensus conference. Int J Color Dis.

[6] Kim NK, Kim YW, Han YD, Cho MS, Hur H, Min BS, et al. Complete mesocolic excision and central vascular ligation for colon cancer: Principle, anatomy, surgical technique, and outcomes. Surg Oncol. 2016;25:252–62. 10.1016/j.suronc.2016.05.009 27566031

[7] West NP, Hohenberger W, Weber K, Perrakis A, Finan PJ, Quirke P (2010). Complete mesocolic excision with central vascular ligation produces an oncologically superior specimen compared with standard surgery for carcinoma of the colon. J Clin Oncol.

[8] Jin G, Tuo H, Sugiyama M, Oki A, Abe N, Mori T (2006). Anatomic study of the superior right colic vein: its relevance to pancreatic and colonic surgery. Am J Surg.

[9] Melich G, Jeong DH, Hur H, Baik SH, Faria J, Kim NK (2014). Laparoscopic right hemicolectomy with complete mesocolic excision provides acceptable perioperative outcomes but is lengthy—analysis of learning curves for a novice minimally invasive surgeon. Can J Surg.

[10] Sonneland J, Anson BJ, Beaton LE (1958). Surgical anatomy of the arterial supply to the colon from the superior mesenteric artery based upon a study of 600 specimens. Surg Gynecol Obstet.

[11] Ignjatovic D, Sund S, Stimec B, Bergamaschi R (2007). Vascular relationships in right colectomy for cancer: clinical implications. Tech Coloproctol.

[12] Hirai K, Yoshinari D, Ogawa H, Nakazawa S, Takase Y, Tanaka K (2013). Three-dimensional computed tomography for analyzing the vascular anatomy in laparoscopic surgery for right-sided colon cancer. Surg Laparosc Endosc Percutan Tech.

[13] Spasojevic M, Stimec BV, Fasel JF, Terraz S, Ignjatovic D (2011). 3D relations between right colon arteries and the superior mesenteric vein: a preliminary study with multidetector computed tomography. Surg Endosc.

[14] Ogino T, Takemasa I, Horitsugi G, Furuyashiki M, Ohta K, Uemura M (2014). Preoperative evaluation of venous anatomy in laparoscopic complete mesocolic excision for right colon cancer. Ann Surg Oncol.

[15] Romano S, Romano L (2010). Utilization patterns of multidetector computed tomography in elective and emergency conditions: indications, exposure risk, and diagnostic gain. Semin Ultrasound CT MR.

[16] Smith CL, Horton KM, Fishman EK (2006). Mesenteric CT angiography: a discussion of techniques and selected applications. Tech Vasc Interv Radiol.

[17] Feng B, Ling TL, Lu AG, Wang ML, Ma JJ, Li JW (2014). Completely medial versus hybrid medial approach for laparoscopic complete mesocolic excision in right hemicolon cancer. Surg Endosc.

[18] Feng B, Sun J, Ling TL, Lu AG, Wang ML, Chen XY (2012). Laparoscopic complete mesocolic excision (CME) with medial access for right-hemi colon cancer: feasibility and technical strategies. Surg Endosc.

[19] Alsabilah J, Kim WR, Kim NK (2017). Vascular structures of the right colon: incidence and variations with their clinical implications. Scand J Surg.

[20] Nesgaard JM, Stimec BV, Bakka AO, Edwin B, Ignjatovic D, RCC study group (2015). Navigating the mesentery: a comparative pre- and per-operative visualization of the vascular anatomy. Color Dis.

[21] Shatari T, Fujita M, Nozawa K, Haku K, Niimi M, Ikeda Y (2003). Vascular anatomy for right colon lymphadenectomy. Surg Radiol Anat.

[22] Menten R, Reding R, Godding V, Dumitriu D, Clapuyt P (2012). Sonographic assessment of the retroperitoneal position of the third portion of the duodenum: an indicator of normal intestinal rotation. Pediatr Radiol.

[23] Halappa V, Zaheer A, Zimmerman SL, Fishman EK (2015). Reversal of superior mesenteric artery and vein in midgut volvulus. Pearls and pitfalls in cardiovascular imaging: pseudolesions, artifacts and other difficult diagnoses.

[24] Sodhi KS, Bhatia A, Saxena AK, Rao KL, Menon P, Khandelwal N (2014). Anticlockwise swirl of mesenteric vessels: a normal CT appearance, retrospective analysis of 200 pediatric patients. Eur J Radiol.

[25] Yada H, Sawai K, Taniguchi H, Hoshima M, Katoh M, Takahashi T (1997). Analysis of vascular anatomy and lymph node metastases warrants radical segmental bowel resection for colon cancer. World J Surg.

[26] Niculescu MC, Niculescu V, Ciobanu IC, Daescu E, Jianu A, Sisu AM (2005). Correlations between the colic branches of the mesenteric arteries and the vascular territories of the colon. Romanian J Morphol Embryol.

[27] Gillot C, Hureau J, Aaron C, Martini R, Thaler G, Michels NA (1964). The superior mesenteric vein, an anatomic and surgical study of eighty-one subjects. J Int Coll Surg.

[28] Yamaguchi S, Kuroyanagi H, Milsom JW, Sim R, Shimada H (2002). Venous anatomy of the right colon: precise structure of the major veins and gastrocolic trunk in 58 cadavers. Dis Colon Rectum.

[29] Ignjatovic D, Spasojevic M, Stimec B (2010). Can the gastrocolic trunk of Henle serve as an anatomical landmark in laparoscopic right colectomy? A postmortem anatomical study. Am J Surg.

[30] McDaniel KP, Charnsangavej C, DuBrow RA, Varma DG, Granfield CA, Curley SA (1993). Pathways of nodal metastasis in carcinomas of the cecum, ascending colon, and transverse colon: CT demonstration. AJR Am J Roentgenol.

[31] Descomps PL (1912). Les veines mésentériques. Anat Physio Norm Pathol Homme Anita.

[32] Falconer CW, Griffiths E (1950). The anatomy of the blood-vessels in the region of the pancreas. Br J Surg.

[33] Van Damme J, Bonte J (1990). Vascular anatomy in abdominal surgery.

[34] Lange JF, Koppert S, van Eyck CH, Kazemier G, Kleinrensink GJ, Godschalk M (2000). The gastrocolic trunk of Henle in pancreatic surgery: an anatomo-clinical study. J Hepato-Biliary-Pancreat Surg.

[35] Spasojevic M, Stimec BV, Dyrbekk AP, Tepavcevic Z, Edwin B, Bakka A (2013). Lymph node distribution in the d3 area of the right mesocolon: implications for an anatomically correct cancer resection. A postmortem study. Dis Colon Rectum.

[36] Tajima Y, Ishida H, Ohsawa T, Kumamoto K, Ishibashi K, Haga N (2011). Three-dimensional vascular anatomy relevant to oncologic resection of right colon cancer. Int Surg.

[37] Garcia-Ruiz A, Milsom JW, Ludwig KA, Marchesa P (1996). Right colonic arterial anatomy. Implications for laparoscopic surgery. Dis Colon Rectum.

[38] Yildirim M, Celik HH, Yildiz Z, Tatar I, Aldur MM (2004). The middle colic artery originating from the coeliac trunk. Folia Morphol (Warsz).

[39] Makomaska-Szaroszyk E, Fiedor P (1989). A rare case of anastomosis between the dorsal pancreatic artery and the middle colic artery. Folia Morphol (Warsz).

